# Immunogenicity and Protective Efficacy of a Live Attenuated H5N1 Vaccine in Nonhuman Primates

**DOI:** 10.1371/journal.ppat.1000409

**Published:** 2009-05-01

**Authors:** Shufang Fan, Yuwei Gao, Kyoko Shinya, Chris Kafai Li, Yanbing Li, Jianzhong Shi, Yongping Jiang, Yongbing Suo, Tiegang Tong, Gongxun Zhong, Jiasheng Song, Ying Zhang, Guobin Tian, Yuntao Guan, Xiao-Ning Xu, Zhigao Bu, Yoshihiro Kawaoka, Hualan Chen

**Affiliations:** 1 Animal Influenza Laboratory of the Ministry of Agriculture and National Key Laboratory of Veterinary Biotechnology, Harbin Veterinary Research Institute, Chinese Academy of Agricultural Sciences, Harbin, People's Republic of China; 2 The International Center for Medical Research and Treatment, Kobe University, Kobe, Japan; 3 MRC Human Immunology Unit, The Weatherall Institute of Molecular Medicine, University of Oxford, United Kingdom; 4 Division of Virology, Department of Microbiology and Immunology, and International Research Center for Infectious Diseases, Institute of Medical Science, University of Tokyo, Tokyo, Japan; 5 Department of Pathobiological Sciences, University of Wisconsin-Madison, Madison, Wisconsin, United States of America; University of Wisconsin-Madison, United States of America

## Abstract

The continued spread of highly pathogenic H5N1 influenza viruses among poultry and wild birds, together with the emergence of drug-resistant variants and the possibility of human-to-human transmission, has spurred attempts to develop an effective vaccine. Inactivated subvirion or whole-virion H5N1 vaccines have shown promising immunogenicity in clinical trials, but their ability to elicit protective immunity in unprimed human populations remains unknown. A cold-adapted, live attenuated vaccine with the hemagglutinin (HA) and neuraminidase (NA) genes of an H5N1 virus A/VN/1203/2004 (clade 1) was protective against the pulmonary replication of homologous and heterologous wild-type H5N1 viruses in mice and ferrets. In this study, we used reverse genetics to produce a cold-adapted, live attenuated H5N1 vaccine (AH/AA*ca*) that contains HA and NA genes from a recent H5N1 isolate, A/Anhui/2/05 virus (AH/05) (clade 2.3), and the backbone of the cold-adapted influenza H2N2 A/AnnArbor/6/60 virus (AA*ca*). AH/AA*ca* was attenuated in chickens, mice, and monkeys, and it induced robust neutralizing antibody responses as well as HA-specific CD4+ T cell immune responses in rhesus macaques immunized twice intranasally. Importantly, the vaccinated macaques were fully protected from challenge with either the homologous AH/05 virus or a heterologous H5N1 virus, A/bar-headed goose/Qinghai/3/05 (BHG/05; clade 2.2). These results demonstrate for the first time that a cold-adapted H5N1 vaccine can elicit protective immunity against highly pathogenic H5N1 virus infection in a nonhuman primate model and provide a compelling argument for further testing of double immunization with live attenuated H5N1 vaccines in human trials.

## Author Summary

H5N1 influenza viruses have caused human infections with more than 60% fatality in 14 countries and may yet be the source of the next pandemic. Therefore, the development of effective vaccines against these viruses is the highest priority for H5N1 pandemic preparedness. A high dosage or adjuvants improve the immunogenicity of H5N1 inactivated vaccines; however, limited production capacity for conventional inactivated influenza virus vaccines could severely hinder the ability to control the spread of H5N1 influenza through vaccination. Here, we generated and tested the efficacy of a cold-adapted, live attenuated H5N1 vaccine in mice and nonhuman primates. We found that the vaccine provided complete protection in these animals against homologous and heterologous H5N1 virus challenge. Since live vaccines require less processing than inactivated vaccines and do not require adjuvants, our study represents a major advance in vaccine development for H5N1 pandemic influenza.

## Introduction

In 1996, a highly pathogenic H5N1 avian influenza virus was detected in geese in China [1]. A year later, a reassortant H5N1 virus caused disease outbreaks in poultry in Hong Kong [2] and was transmitted to humans, infecting 18 people, six of whom died [3],[4]. Beginning in late 2003, outbreaks of H5N1 influenza A virus infection appeared among poultry, and wild birds in numerous countries in Asia and subsequently were reported in Europe and Africa (Office International des Epizooties [OIE]; http://www.oie.int). Despite substantial efforts to control the infection in poultry, H5N1 viruses have continued to evolve and spread, producing human infections in 14 countries, with 236 of the 372 confirmed cases proving fatal (World Health Organization [WHO]; http://www.who.int). The emergence of H5N1 viruses resistant to adamantanes and oseltamivir [5],[6],[7] has raised serious concerns over the ability of current antiviral agents to prevent global influenza outbreaks. Thus, the development of an effective vaccine has assumed the highest priority in preparedness for an H5N1 influenza pandemic.

H5N1 inactivated vaccines can induce functional and cross-reactive antibodies that protect ferrets or nonhuman primates from H5N1 infection [8], and have been shown to be safe and tolerable in human trials [9],[10],[11]. With the addition of adjuvants, such vaccines induce antibody titers that are known to provide protection against seasonal influenza in humans [11], however, the antibody level considered to be protective was based on findings in humans who had likely been exposed to the seasonal human virus and thus were “preimmunized”. Because the vast majority of humans have not been exposed to highly pathogenic H5N1 viruses, it is still unknown whether the level of antibody known to be protective against seasonal human influenza virus infection would also be effective against H5N1 viruses. Additionally, while humoral immunity is effectively induced by the inactivated vaccines, the cellular immune response is not [12]. This deficit has raised concern because of indications that the cellular immune response may play a significant role in protection against H5N1 infection [12].

The cold-adapted (*ca*) influenza virus A/Ann Arbor/6/60 (AA) (H2N2) has been developed as a live attenuated vaccine seed virus that exhibits cold-adaptation, temperature-sensitive (*ts*), and attenuation (*att*) phenotypes which are specified by mutations in the internal genes. Reassortant H1N1 and H3N2 human influenza A viruses with the six internal gene segments of the AA*ca* virus have been repeatedly demonstrated to bear these phenotypes and extensive evaluation in humans has proven them to be attenuated and safe as live virus vaccines (reviewed in [13]–[15]). In previous studies, live attenuated H5N1 vaccines generated by reverse genetics and comprising internal genes of the AA*ca* virus and the HA and NA genes derived from earlier H5N1 influenza viruses were proved to be safe in mice and ferrets, and to protect these animals from death against different H5N1 viruses challenges [16],[17]. In this study, we produced three live attenuated, *ca* H5N1 viruses, using reverse genetics, that contain the HA and NA genes of H5N1 viruses isolated at different times and from different species in China. After *in vitro* and in *vivo* analyses, one of the cold-adapted virus that contains the HA and NA genes from a recent H5N1 virus, A/Anhui/2/2005 (AH/05) (clade 2.3), was selected for immunogenicity and efficacy testing in mice and nonhuman primates.

## Results

### Generation and characterization of H5N1 cold-adapted reassortant viruses

We constructed three H5N1 reassortant virus by reverse genetics [18],[19],[20], using the published sequences of the low pathogenic A/Ann Arbor/6/60 ca virus (AA*ca*) [21] to generate all of the genes encoding the internal proteins. The HA and NA genes were from the highly pathogenic H5N1 influenza viruses, including A/goose/Guangdong/1/1996 (GS/GD/96) (clade 0), A/chicken/Shanxi/2/2006(CK/SX/06), a virus isolated from chickens in northern Chine in 2006, and A/Anhui/2/2005 (AH/05) (clade 2.3), which was isolated from a human in China (WHO; http://www.who.int). The multiple basic amino acids at the HA cleavage site, a major virulence motif for H5N1 influenza viruses, were replaced with those found at the HA cleavage site of a nonpathogenic avian influenza virus, as previously described [16],[22]. The cold-adapted (*ca*) and temperature-sensitive (*ts*) phenotypes of the resultant viruses GSGD/AA*ca*, CKSX/AA*ca*, and AH/AA*ca*, attributable to the internal genes of AA*ca*, were also confirmed as described previously (data not shown). The *ca* reassortant viruses did not cause disease or death in chickens upon intranasal or intravenous administration, while all of the three wild-type H5N1 viruses are lethal to chickens (Table 1).

**Table 1:** Attenuation phenotype of the recombinant AH/AA*ca* virus in chickens.

Virus	Observations after intranasal inoculation^a^						Observations after intravenous inoculation^b^		
	Virus shedding on day 3 (log_10_ EID_50_)		Virus shedding on day 5 (log_10_ EID_50_)		No.SC./total	No.sick deaths/total^c^	No.sick/deaths/total	No.SC/total	IVPI
	Oropharyngeal	Cloacal	Oropharyngeal	Cloacal					
GSGD/AA*ca*	<	<	<	<	0/6	0/0/6	0/0/10	2/10	0
GS/GD/96	3.2±0.3	2.4±0.8	2.4±0.3	2.8±0.6	NA	0/6/6	1/9/10	1/1	2.1
CKSX/AA*ca*	<	<	<	<	0/6	0/0/6	0/0/10	3/10	0
CK/SX/06	3.2±0.6	2.1±0.4	2.5	3.0	NA	0/6/6	0/10/10	NA	2.8
AH/AA*ca*	<	<	<	<	0/6	0/0/6	0/0/10	2/10	0
AH/05	2.4±0.6	1.7±0.6	1.4±0.1	1.8±0.1	NA	0/6/6	0/10/10	NA	2.5

a Six-week-old specific-pathogen free chickens were inoculated i.n. with 10^6^ EID_50_ of virus in a 0.1-ml volume. Swabs were collected from the birds on day 3 and day 5 postinoculation and titrated in eggs. <, virus not detected. data are means±standard deviations. No. sick/deaths/total; numbers of chickens that were sick and died, as well as the total number of chickens during the observation period. Birds that showed disease signs, such as depression and ruffled feathers, but recovered at the end of the observation were counted as sick animals. SC/total, number of chickens that seroconverted out of the total number of chickens at the end of the 2-week observation period. NA, all birds died by the end of the observation period and thus could not be studied for sera conversion.

b Six-week-old white Leghorn chickens housed in high-efficiency particulate air-filtered isolators were inoculated i.v. with 0.2 ml of a 1∶10 dilution of bacterium-free allantoic fluid containing virus for intravenous pathogenicity index (IVPI) testing, based on recommendations of the Office International Des Epizooties.

c Only animals that showed disease signs but recovered by the end of the observation period were identified as sick birds.

### Replication and immunogenicity of the reassortant viruses in mice

We evaluated the replication of GSGD/AA*ca*, CKSX/AA*ca*, and AH/AA*ca*, in mammals, using the BALB/c mouse. Groups of 6-week-old female BALB/c mice were inoculated intranasally with 10^6^ EID_50_ (dose required to infect 50% of eggs) of the reassortant viruses or their wild-type H5N1 viruses. Three mice in each group were killed on day 3 postinoculation (p.i.) and their organs were collected for virus titration. As previously reported [1], the replication of the wild-type GS/GD/96 virus was not detected in any organs on day 3 p.i.; however, the *ca* virus GSGD/AA*ca* was detected in the nasal turbinate of the inoculated mice at this timepoint (Table 2). The wild-type CK/SX/06 virus replicated in the nasal turbinate and lung, while the replication of the reassortant *ca* virus CKSX/AA*ca* was not detected in any organs (Table 2). The wild-type AH/05 replicated systemically with high virus titers in all of the organs examined. The replication of AH/AA*ca*, by contrast, was restricted to the respiratory system. Even in lung, the AH/AA*ca* titer was significantly lower than that for AH/05 (Table 2). The reassortant viruses, as well as the wild-type GS/GD/96 and CK/SX/06 viruses did not kill any mice at the highest inoculation dose, whereas the AH/05 virus killed mice at a very low dosage (MLD_50_ = 1.5 log_10_EID_50_) (Table 2).

**Table 2:** Replication of the H5N1 *ca* reassortants and the wild-type H5N1 viruses in mice.

Virus	Mean virus titer (log_10_ EID_50_/ml±SD) on day 3 post inoculation in:^a^					MLD_50_ (log_10_EID_50_)
	Turbinate	Lung	Spleen	Kidney	Brain	
GSGD/AA*ca*	3.1±0.6	<	<	<	<	>7.5
GS/GD/96	<	<	<	<	<	>8.0
CKSX/AA*ca*	<	<	<	<	<	>7.8
CK/SX/06	1.8±0.6	4.4±0.8	<	<	<	>6.5
AH/AA*ca*	3.8±0.1	3.7±0.5^b^	<	<	<	>7.2
AH/05	4.7±0.9	6.5±1.2	2.7±0.8	2.0±0.5	2.6±1.0	1.5

a Six-week-old BALB/c mice (3 per group), inoculated intranasally with 10^6^ EID_50_ of the indicated virus in a 50-µl volume, were killed on day 3 postinoculation and their organs were collected for virus titration in eggs. <, no virus was isolated from that sample.

b P value was<0.01 compared with the titers in the corresponding organs of the AH/05-inoculated mice.

We then evaluated the immunogenicity of the three reassortant *ca* H5N1 viruses in mice. Four weeks after the first intranasal immunization of GSGD/AA*ca*, hemagglutinin inhibition (HI) antibody against the homologous virus GS/GD/96 was not detected, but the neutralization (NT) antibody titers was 320. After the second vaccination, the HI and NT antibodies increased sharply to titers of 80 and 1067, respectively (Table 3). HI and NT antibodies in the CKSX/AA*ca*-inoculated mice were not detected even after the second vaccination (Table 3). However, both HA and NT antibodies were detected in mice after one vaccination of the AH/AA*ca* virus, and the mean titers increased sharply after the second vaccination (Table 3). These results indicate that the AH/AA*ca* virus induced a better immune response than the other two *ca* reassortant viruses. Therefore, we selected the AH/AA*ca* virus for further vaccine efficacy investigations in mice and monkeys.

**Table 3:** Antibody response induced by the H5N1 *ca* reassortant viruses in mice.

Virus	Mean antibody titers in mice					
	Pretest		Dose 1		Dose 2	
	HI	NT	HI	NT	HI	NT
GSGD/AA*ca*	<5	<10	<5	320	80	1067
CKSX/AA*ca*	<5	<10	<5	<10	<5	<10
AH/AA*ca*	<5	<10	26.7	533	480	1573

Group of five six-week-old BALB/c mice were inoculated with two dose, in a 4 week interval, of 10^6^ EID_50_ of the indicated H5N1 *ca* reassortant virus. Four weeks after dose 1 or dose 2, sera were collected for determining the HI and NT antibodies using the homologous wild type H5N1virus.

### Protective efficacy of the AH/AA*ca* vaccine in mice

**Figure 1:**
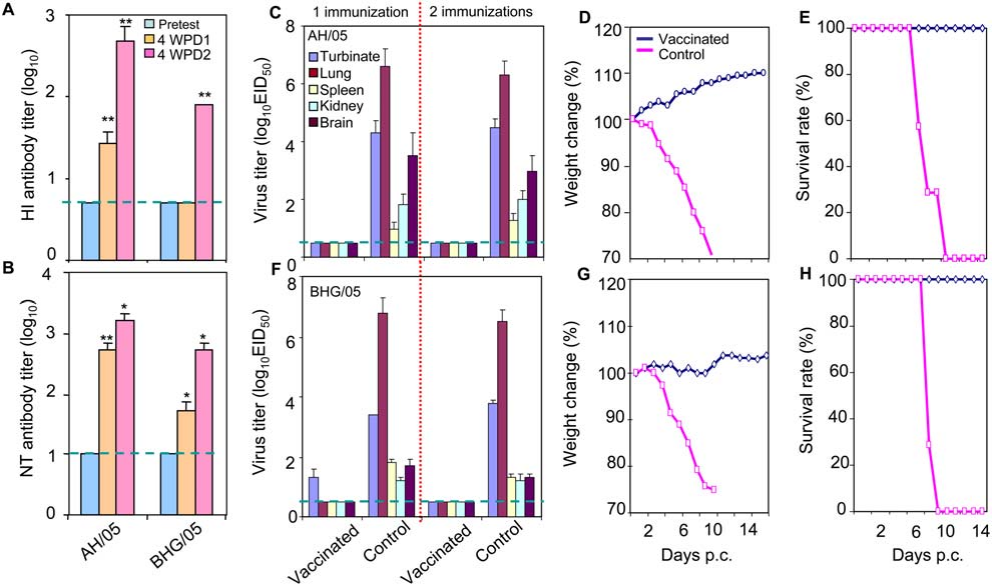
Vaccine efficacy of the AH/AA*ca* virus in mice. (A) HI and (B) NT antibody responses to homologous (AH/05) and heterologous (BHG/05) viruses after intranasal vaccination with 10^6^ EID_50_ of AH/AA*ca* in a 50-µl volume. Serum samples were collected on day 0 prevaccination (blue), 4 weeks after the first (orange) and 4 weeks after the second vaccination (pink). Asterisks indicate a statistically significant difference from antibody titers measured at the preceding time point: **, P<0.01, *, P<0.05. (C–H) Protective efficacy against challenge with the AH/05 (C–E) or BHG/05 virus (F–H). Weight changes (D and G) and survival rates (E and H) are shown only for the groups that were immunized once. The data in panels A–C and F are reported as means±s.d.; the dashed blue lines in these panels indicate the lower limit of detection. p.c., postchallenge.

We first evaluated the protective efficacy of the AH/AA*ca* vaccine in mice. By 4 weeks after the first intranasal immunization of 10^6^ EID_50_ of AH/AA*ca*, the mean±s.d. titers of HI and NT antibodies against the homologous AH/05 virus had increased significantly over the pretest values (P<0.01) (Figure 1A and 1B). They also rose sharply after the second vaccination (P<0.01 [HI], P<0.05 [NT]) (Figure 1A and 1B). Antibodies to the heterologous A/bar-headed goose/Qinghai/3/05 (BHG/05) (clade 2.2) virus were either undetectable (HI) or increased (NT) after the first vaccination, rising to 80±23 (HI, P<0.01) and 533±184 (NT, P<0.05) after the second immunization (Figure 1A and 1B).

Four weeks after vaccination, we challenged the mice intranasally with a lethal dose (10^2^ LD_50_) of two different H5N1 viruses, AH/05 and BHG/05, whose genetic and antigenic properties are different from those of the AH/05 virus. Three mice were killed on day 3 postchallenge, and their organs were collected for virus titration; the remaining seven mice in each group were observed for 2 weeks. As shown in Figure 1C–1E, mice were completely protected from homologous AH/05 virus challenge in both the single- and two-vaccination groups. Virus was not detected in any of the organs tested, and the mice remained healthy over the 2 weeks of observation (no weight loss). By contrast, the virus replicated systemically and was detected in all of the test organs in unvaccinated mice, with death occurring between 6 and 10 days postchallenge. In mice challenged with BHG/05, virus was detected at low titers (<2 log_10_ EID_50_ g^−1^) in the nasal turbinates of animals that had received a single dose of vaccine, but was undetectable in the organs from mice that were vaccinated twice (Figure 1F). All of the vaccinated mice remained healthy during the 2-week observation period, whereas the virus replicated systemically and killed all of the mice within 10 days postchallenge in the unvaccinated group (Figure 1G and 1H).

### Humoral and cellular immune responses to the AH/AA*ca* vaccine in rhesus macaque

To assess the immunogenicity of the AH/AA*ca* reassortant virus in a rhesus macaque (*Macaca mulatta*) model, we inoculated 2- to 3-year-old female animals (n = 8, Vaccinated 1–8, V1–V8) intranasally with 10^7^ EID_50_ of the AH/AA*ca* virus in a 1-ml volume, twice, at a 4-week interval. A control group (n = 8, Control 1–8, C1–C8) received the same volume of phosphate-buffered saline (PBS). Serum was collected from each animal at 4 weeks after the first vaccination (week postvaccination dose, wpd1) and at 2 weeks after the second vaccination (wpd2). Peripheral blood mononuclear cells (PBMCs) of the monkeys were isolated at different times for detection of a T-cell immune response using an H5-HA specific IFN-γ ELISPOT assay. Intranasal inoculation of the AH/AA*ca* virus was not associated with any adverse events (not shown).

**Figure 2:**
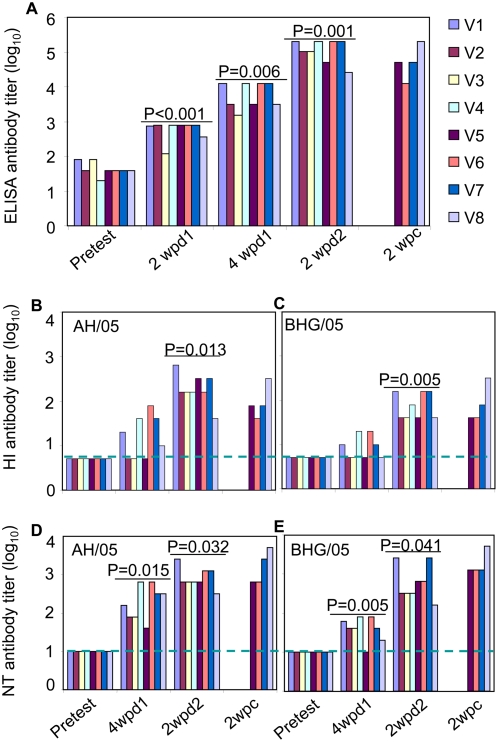
Antibody responses of nonhuman primates. (A) H5N1-specific antibody levels assessed by ELISA. Hemagglutination-inhibition (HI) antibody to AH/05 (B) and BHG/05 (C) with chicken erythrocytes, and microneutralization (NT) antibody to the AH/05 (D) and BHG/05 (E) viruses. The HI antibody titers with horse erythrocytes were 4- to 8-fold higher than those with chicken erythrocytes (data not shown). Titers are reported for individual vaccinated animals. Blue dashed lines indicate the lower limit of detection. Wpd1/2, week postvaccination dose 1 or 2; wpc, week postchallenge. The P values indicate the antibody titers with a significant increase from the preceding time point. In the control animals, the HI and NT antibodies at all time points are the same as the pretested values, only the ELISA antibody titers, ranged 400–800, were detected at two weeks postchallenge (data not shown).

**Figure 3:**
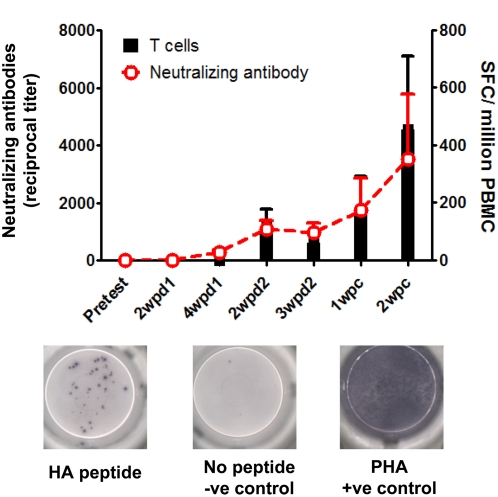
T cell responses against HA antigens in rhesus macaques and its relationship to the neutralizing antibody. T cell responses in the PBMC samples were measured by overlapping peptides and ex-vivo IFN-γ ELISPOT assay. Data shown are the mean numbers ±s.d. of spot-forming cells (SFC) per 10^6^ PBMCs to HA peptide pools in the vaccinated animals at different time points after vaccination and challenge. Neutralizing antibody in the serum samples was measured by the microneutralization method using AH/05 virus isolate, data shown are mean titers ±s.d. The T cell response and the neutralizing antibody were not detected in the control monkeys at all time points tested (data not shown).

As shown in Figure 2A, each of the vaccinated macaques had a detectable antibody response by ELISA (enzyme-linked immunosorbent assay) at 2 weeks after the first inoculation, with the titer ranging from 120–780 (median, 760), and the titers increased sharply at 4 weeks after the first inoculation, with titers ranging from 1520 to 12780 (median, 7640). HI and NT antibodies were not detectable from the animals at 2 weeks after the first inoculation (data not shown). Five of these animals developed HI antibodies to the AH/05 virus (titers, range of 10–80, median, 40), and all had NT antibodies to this virus (range of titers, 40–640, median 240) (Figure 2B and 2D) at 4 weeks after the first inoculation. Antibody levels in the vaccinated animals increased significantly after the second vaccination (Figure 2). Eight animals had detectable HI antibody to AH/05 (range of titers, 40–640; median, 160) at 2 weeks after the second vaccination, while NT and ELISA antibody titers reached 320–2560 and 25,118–199,526, respectively, at this interval (Figure 2A, 2B, and 2D). Overall, the HI and NT antibody titers against the heterologous virus BHG/05 (Figure 2C and 2E) were 2- to 4-fold lower than those against the AH/05 virus. T cell responses to the HA protein were not detected at 4 weeks after the first immunization. However, the HA-specific T cell responses could be detected at 2 weeks after the second immunization in all vaccinated macaques (Figure 3). Interestingly, 11 of the 13 T cell HA-specific peptides identified in samples from eight macaques represented CD4+ T cells (Table 4), suggesting this T cell subset may have played a role in the generation of antibodies against H5N1 epitopes. The HI and NT antibodies and the T cell response of the control animals at all time points before challenge are the same as the pretested values.

**Table 4:** T cell peptides and their corresponding T cell subsets against H5 HA in monkeys vaccinated with AH/AA*ca*.

HA peptide^a^		T cell response to the peptide		
Peptide sequence	Amino acid position	SFC/million PBMC^b^	No. animals responding	T cell subset^c^
VKSDQICIGYHANNSTEQV	14–32	375	1/8	CD4+
MEKNVTVTHAQDILEKTH	45–53	500	1/8	CD4+
NPMCDEFINVPEWSYIV	79–96	200	1/8	CD4+
LCYPGNFNDYEELKHLL	105–121	250–332	2/8	CD4+
NDYEELKHLLSRINHFEK	111–129	267	1/8	CD8+
PKSSWSDHEASSGVSSA	134–150	186	1/8	CD8+
SFFRNVVWLIKKNNTY	158–173	349–476	2/8	CD4+
NDAAEQTKLYQNPTTYI	198–204	50–66	3/8	CD4+
KLYQNPTTYISVGTSTL	214–221	625	1/8	CD4+
VPKIATRSKVNGQSGRM	226–242	143–169	3/8	CD4+
ILKPNDAINFESNGNFIA	248–265	375	1/8	CD4+
GWQGMVDGWYGYHHSNEQ	358–375	175	1/8	CD4+
LKREEISGVKLESIGTY	513–529	188	1/8	CD4+

a Sequences were based on the HA gene of A/Anhui/1/05 virus.

b The numbers of IFN-γ–positive T lymphocytes in PBMC samples expressed as spot-forming cells (SFC)/million PBMCs were determined by HA overlapping peptides and IFN–ELISPOT assay.

c T cell subset of the HA-specific T cells in PBMC samples was further determined by cell depletion using magnetic beads against CD8 and IFN-γ ELISPOT assay.

### Protective efficacy of AH/AA*ca* in monkeys against homologous and heterologous H5N1 virus challenge

Three weeks after the second vaccination, animals in each group were challenged with an intratracheal inoculation of 10^6^ EID_50_ of AH/05 virus (n = 4) or BHG/05 virus (n = 4) in a 3-ml volume. Three days later, two animals from each group were euthanized, and different parts of the respiratory system were collected for virus titration and histologic and immunohistochemical studies. The remaining animals were observed and euthanized on day 15 postchallenge.

**Figure 4:**
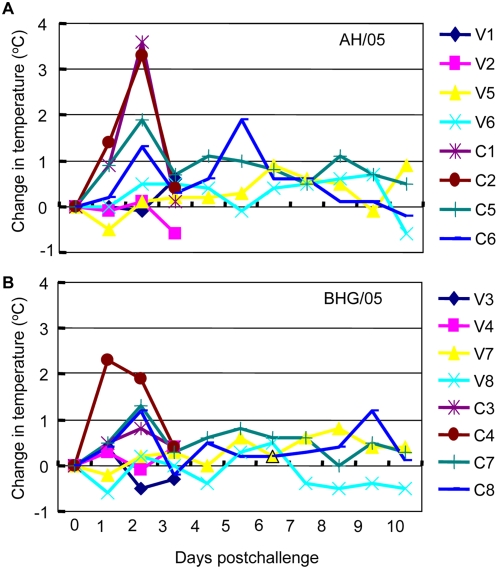
Body temperature of nonhuman primates after challenge with AH/05 virus. Change in body temperature in nonhuman primates after challenge with AH/05 virus (A) or BHG/05 virus (B). Changes were calculated by subtracting the mean temperature 3 days before challenge from the temperature recorded on the indicated day.

The control animals showed disease symptoms after challenge. All eight control animals became anorexic on day 1 postchallenge, and completely lost their appetites for two days. Four were euthanized on day 3 postchallenge, while the remaining four animals gradually recovered on days 4 and 5 postchallenge. Four control animals challenged with AH/05 and three challenged with BHG/05 developed fever within the first 2 days postchallenge (Figure 4A and 4B). By contrast, the vaccinated animals remained healthy during the 15 days of observation post-challenge with either the AH/05 or BHG/05 virus. The appetites and body temperatures of the vaccinated animals were unchanged during this period (Figure 4A and 4B).

In the vaccinated animals, the HI and NT antibody titers measured at 2 weeks postchallenge were approximately the same as those recorded at 2 weeks after the second vaccination (Figure 2B–2E). HA-specific T cell responses increased on week 1 after challenge, and the peak response was detected at 2 weeks postchallenge (Figure 3). While in the control animals, although the titers of antibodies measured by ELISA reached 400–800, the HI and NT antibody, and the HA-specific T cell responses were not detected at 2 weeks after challenge (data not shown).

**Figure 5:**
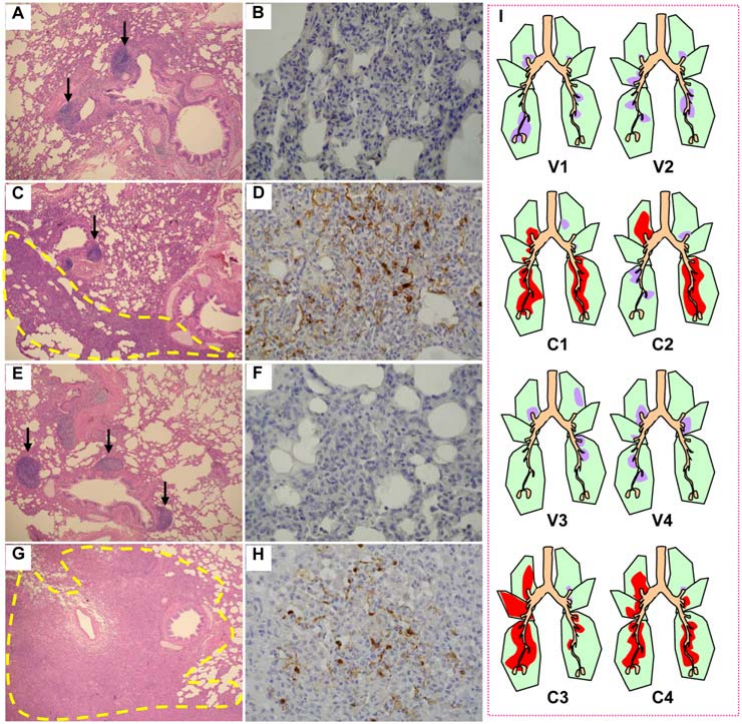
Vaccine efficacy in nonhuman primates assessed on the basis of lung lesions. Eight macaques were euthanized on day 3 postchallenge with AH/05 virus (A–D) or BHG/05 virus (E–H). Vaccinated animals (A,B,E,F) had less extensive bronchopneumonia (i.e., smaller foci of consolidation) than did unvaccinated animals (C,D,G,H). The vaccinated animals also showed prominent peribronchial lymph follicles (a, e; arrows), and their consolidated lung areas lacked viral antigen-positive cells (B,F). By contrast, the unvaccinated animals had lung lesions of moderate size with a wide consolidated area (C,G; outlined by yellow dashes), smaller and less abundant peribronchial lymph follicles (C,G; arrows), and pneumonic lesions containing many antigen-positive cells (D,H; brown pigment). (I) Schematic diagrams indicating distribution of pathologic lesions in the lungs of animals vaccinated and challenged with AH/05 (V1 and V2); nonvaccinated and challenged with AH/05 (C1 and C2); vaccinated and challenged with BHG/05 (V3 and V4); and nonvaccinated and challenged with BHG/05 (C3 and C4). In vaccinated animals, scant-to-moderate bronchopneumonia was present in each lobe, but viral antigens were not detected in the lesions (V1, V2, V3, and V4; purple). By contrast, more severe bronchopneumonia was observed in nonvaccinated macaque lungs (C1, C2, C3, and C4). Moreover, viral antigens were prominent in the pneumonic lesions in the most affected lung lobes (C1, C2, C3, and C4; red). One lung lobe was entirely affected by pneumonia after infection with the BHG/05 virus (C3). Purple, bronchopneumonia without viral antigen; red, bronchopneumonia with viral antigen.

Lung tissue from four vaccinated macaques euthanized on day 3 postchallenge with either BHG/05 or AH/05 lacked macroscopic lesions, had only mild-to-moderate bronchopneumonia with prominent peribronchiolar lymph follicles apparent on microscopic observation, and were free of detectable viral antigen (Figure 5A, 5B, 5E, 5F, and 5I). A spectrum of macroscopic lesions—including congestion, exudation, and consolidation—were observed in the lung lobes of two unvaccinated control animals challenged with the BHG/05 virus (C3 and C4) and one challenged with the AH/05 virus (C1). Only prominent swelling of the lymph nodes and tonsil were seen in another control animal (C2) challenged with the AH/05 virus. Moderate-to-severe bronchopneumonia with prominent viral antigen expression was a characteristic finding in the nonvaccinated animals (Figure 5C, 5D, 5G, 5H, and 5I; also Table 5).

**Table 5:** Pathological lesions and antigen distribution in tissues of nonhuman primates infected with H5N1 viruses.

Challenge virus	Animal	Vaccination with AH/AA*ca*	Tra	Bro(R)	Bro(L)	Right lungs			Left lungs			Tonsil	TBLN
						Upper	Middle	Lower	Upper	Middle	Lower		
AH/05	V1	+	−/−b	−/−	−/−	+/−	−/−	++/−	+/−	−/−	+/−	−/−	−/−
	V2	+	−/−	−/−	−/−	+/−	+/−	+/−	+/−	−/−	++/−	−/−	−/−
	C1	−	−/−	−/−	−/−	++/+	+/+	++/++	+/−	+/−	++/++	−/−	−/−
	C2	−	−/−	−/−	−/−	++/+	−/−	+/−	+/−	−/−	++/−	−/−	−/−
BHG/05	V3	+	−/−	−/−	−/−	+/−	−/−	−/−	+/−	−/−	+/−	−/−	−/−
	V4	+	−/−	−/−	−/−	+/−	+/−	+/−	−/−	+/−	−/−	−/−	−/−
	C3	−	−/−	−/−	−/−	+/+	+++/++	++/++	+/−	+/−	+/+	−/−	−/−
	C4	−	−/−	−/−	−/−	++/++	++/++	++/++	+/−	+/+	++/++	+/+	−/−

Animals were vaccinated twice (4-week interval) with AH/AA*ca* and challenged with AH/05 or BHG/05 virus. Tissues for pathological examination were collected 3 days after viral challenge. Tra, trachea; Bro, Bronchus; TBLN, tracheobronchial lymph node.

b Pathological lesions/viral antigens. −, no pathological change/antigen. +, limited pathological change/antigen. ++, moderate pathological change/antigen. +++, severe pathological change/abundant antigen.

**Figure 6:**
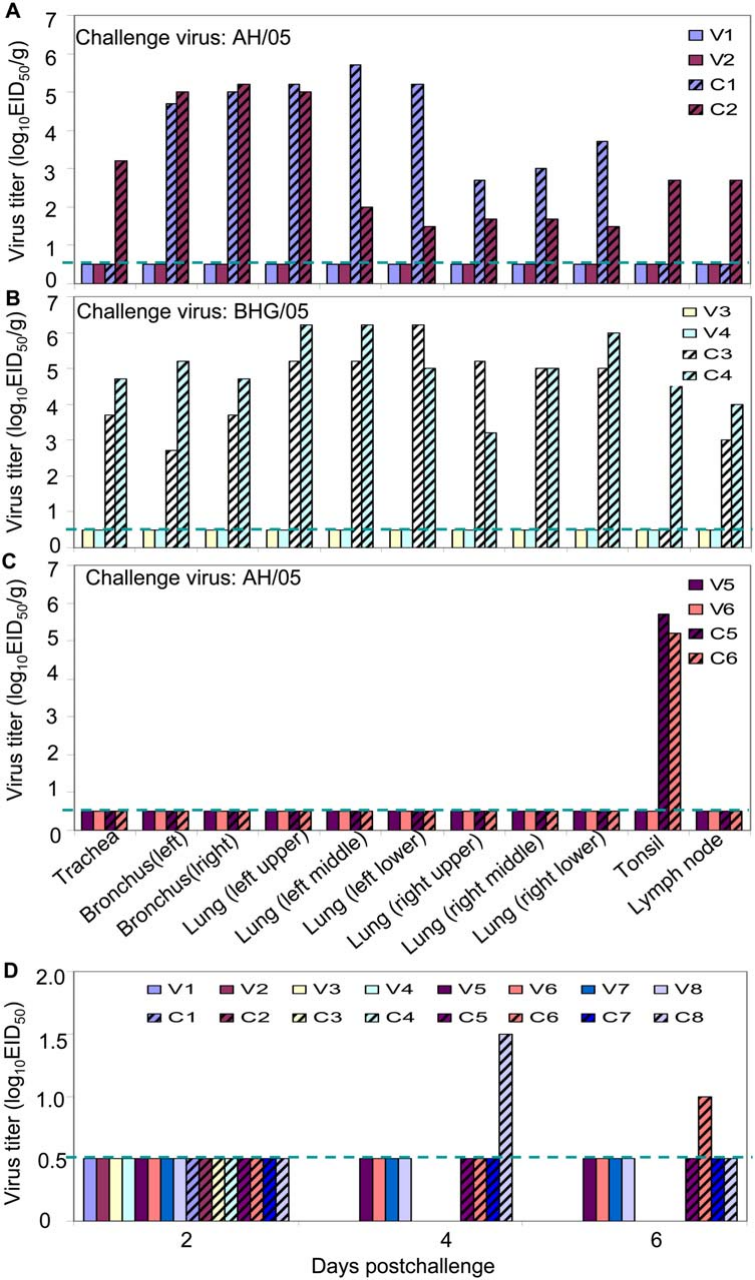
Viral replication in nonhuman primates. Virus titers were determined in embryonated eggs injected with tissue homogenates on day 3 (A,B) and day 15 (C) postchallenge with AH/05 virus or BHG/05 virus. Virus was not detected in tissues harvested day 15 postchallenge from animals challenged with the BHG/05 virus (data not shown). Titers are reported for tissues from individual animals, as log_10_ EID_50_/g tissue. The dashed blue lines indicate the lower limit of detection. (D) Nasal swabs were collected from all living animals on days 2, 4, and 6 postchallenge for virus isolation in eggs.

Virus was not isolated from any of the organs tested in the four vaccinated animals challenged with either the AH/05 or BHG/05 virus (Figure 6A and 6B), but was found at high titers in the trachea, bronchus, lung, lymph nodes, and tonsil of the four unvaccinated animals on day 3 postchallenge (Figure 6A and 6B). Among the eight macaques euthanized on day 15 postchallenge, virus was isolated from tonsil of the two control animals challenged with AH/05 virus (C5 and C6) at titers of 5.3 and 5.7 log_10_EID_50_/g, respectively (Figure 6C), but not from either of the two vaccinated animals. Virus was recovered at a low titer from the nasal swab of a single macaque (C8) on day 4 postchallenge and of another (C6) on day 6 postchallenge (Figure 6D).

## Discussion

We generated a reassortant H5N1 cold-adapted virus, AH/AA*ca*, using reverse genetics in Vero cells, and evaluated its immunogenecity and efficacy as a live attenuated vaccine. The virus retained both the *ca* and *ts* phenotypes of the AA*ca* virus, and was attenuated in chickens, mice and monkeys. After a single immunizing dose, the vaccine induced strong HI and NT antibody responses to H5 influenza virus in mice and protected them from homologous and heterologous H5N1 virus challenges. Most importantly, after two immunizations, the vaccine induced both humoral and T cell immune response in nonhuman primates and completely protected these animals from challenge with either homologous or heterologous H5N1 virus. Our results warrant human testing of the AH/AA*ca* virus as a candidate live attenuated pandemic vaccine for use against H5N1 influenza virus.

The H5N1 viruses are divided into ten distinct phylogenetic clades (0–9) based on their HA genes. Those associated with human infection are all from either clade 1, representing viruses isolated mainly from patients in Thailand and Vietnam, or clade 2, in which the viruses have been further divided into different subclades [23]. The clade 2.1 viruses are circulating only in Indonesia, while the clade 2.2 and 2.3 viruses continue to infect poultry and humans in multiple countries, posing severe threats to public health [23]. The H5N1 pandemic vaccines evaluated to date are all based on clade 1 [9]–[11],[17] or calde 0 viruses [16],[17], and their efficacy against clade 2 viruses is quite limited [17]. The AH/AA*ca* vaccine we described provides complete protection against challenge with viruses from both clades 2.2 and 2.3; moreover, the monkey antisera induced by AH/AA*ca* cross reacted well with clade 1 virus as in an HI test (data not shown).

The H5N1 viruses, AH/05 and BHG/05, replicate in multiple organs in mice after intranasal inoculation (Table 2), however, our preliminary studies indicate that these viruses could not replicate in monkeys after intranasal inoculation, but they replicate efficiently in the respiratory system and caused severe pneumonia upon intratracheal inoculation, as has been seen with human patients [24]. This is why we challenged the monkeys with intratracheal inoculation instead of the intranasal inoculation. Despite introduction of the challenge virus into the trachea, a preferred site of replication for H5N1 viruses, the intranasal immunization of the AH/AA*ca* vaccine provided complete protection to animals, further demonstrating the efficacy of this live vaccine.

In a previous study, Suguitan et al [17] found differences in the immunogenicity of three H5N1 live attenuated viruses. The vaccine that contains the HA and NA genes from a clade 1 virus (A/VN/1203/2004) was poorly immunogenic in mice, and was not able to prevent the replication of the challenge viruses in lungs and turbinates of mice (even two doses of the vaccine were not able to prevent the replication of the challenge virus in ferrets). That vaccine was also poorly immunogenic in humans (personal communication from Dr. Subbarao). We also generated different H5N1 live attenuated viruses with the surface genes derived from different viruses that had been isolated in China. We found that these reassortants possessed diverse replicative abilities and induced varied antibody responses in mice; only the AH/AA*ca* vaccine proved highly immunogenic in both mice and monkeys. These results suggest that the immunogenicity of H5N1 live attenuated vaccine largely depends on the HA and NA genes, emphasizing the need for careful selection of donor viruses when preparing vaccines for a likely H5N1 influenza pandemic.

Although the precise responses that must be induced to protect against H5N1 infection in humans are unknown, animal studies indicate a central role for the cellular immune response [12]. Thus, in the face of a pandemic, a vaccine that elicits cellular immunity could be valuable in reducing the severity of disease and mortality, if not in providing complete protection from infection [25]. Moreover, a vaccine that induces a cellular immune response could increase the likelihood of generating broadly cross-reactive responses that may be effective against multiple virus strains. Ruat et al [8] reported that inoculation of two doses of inactivated vaccine (containing 30 µg of HA) with adjuvants in monkeys could induce functional antibody and protect the animals from a homologous H5N1 virus challenge, but that vaccine did not induce any detecable cellular immune response. In the present study, the HI antibody titers induced by two doses of the AH/AA*ca* vaccine in monkeys were higher than those achieved with two doses of the inactivated vaccine in the study of Ruat et al [8]. Two doses of AH/AA*ca* vaccine also induced strong T cell responses, which may play an important role in the sterile protection of monkeys after H5N1 influenza virus challenge.

Whether cold-adapted, live attenuated H5N1 vaccines would be sufficiently immunogenic to merit their widespread use during a pandemic remains unclear, the use of live influenza H5N1 vaccines before a pandemic would be difficult to justify, as this strategy would introduce a new HA gene into human populations. Nonetheless, considering the efficacy shown by our vaccine in nonhuman primates, we suggest that it has strong potential as an effective H5N1 virus countermeasure, warranting further evaluation in humans.

## Materials and Methods

### Viruses

The H5N1 virus AH/05 was isolated from the tracheal secretion of a patient with lethal outcome from Anhui province in China in 2005 [26],[27], the BHG/05 virus and the GS/GD/96 viruses were isolated from a bar-headed goose and a goose, respectively, as described previously [1],[28], and the CK/SX/06 virus was isolated from a chicken in northern China in 2006. Virus stocks were propagated in specific-pathogen free chicken embryos or MDCK cells. The PB2, PB1, PA, NP, M and NS genes of the AA*ca* virus were synthesized (Jinsite Biotechnology, www.jinsite.com.cn) and inserted into the viral RNA-mRNA bidirectional expression plasmid pBD as described previously [29]. The HA and NA genes of the AH/05 virus were amplified by RT-PCR and inserted into pBD; the region that encodes basic amino acids at the HA cleavage site of the plasmid was modifed to encode the amino acid sequence corresponding to the sequence in the avirulent virus HA as described previously [16],[22]. AH/AA*ca* virus was generated by reverse genetics [18],[19],[20]. Virus stock was propagated in specific pathogen-free chicken eggs.

### Phenotypic analysis of the ca reassortant viruses

The *ca* and *ts* phenotype and the replication of AH/AA*ca* in chickens were tested as previously described [30].

### Detection of antibodies

Serum antibody against H5N1 influenza virus was detected by IgG ELISA essentially as described by DiNapoli et al [31] using 400 ng of AH/AAca virus, which was grown in eggs and purified by ultracentrifugation though a 30% sucrose cushion, to coat the 96-well Immulon 1B plates (Dynex Technologies, Inc., www.dynextechnologies.com). The pretest ELISA values (range 20–80) are the background; which have been subtracted from the experimental values of the monkeys. Sera were treated with Vibrio cholerae (Denka-Seiken, www.denka-seiken.co.jp) receptor-destroying enzyme before being tested for the presence of HI antibody with 0.5% (V/V) chicken erythrocytes. The antigens used were homologous wild-type H5N1 virus or BHG/05 virus. The NT antibody titers were tested in MDCK cells with heat-inactivated sera collected from mice or animals. HI and NT antibody titers were transformed into log_10_ titers for the calculation of mean±s.d. values.

### IFN-γ ELISPOT assay

The frequencies of PBMC-derived T lymphocytes that released IFN-γ upon restimulation with H5 HA-derived peptide pools were determined by an ELISPOT assay, using a Mabtech kit according to the manufacturer's instructions. Briefly, thawed PBMC samples, isolated by Lymphoprep density gradient centrifugation (Axis-Shield, www.axis-shield.com), were incubated without peptides at 37°C in 5% CO_2_ for 4 h. After washing, they were incubated for 20 h at a concentration of 20 µg/ml with peptides (18-mer overlapped by 10 amino acids; Sigma, www.sigmaaldrich.com) generated from a human H5N1 isolate (A/Anhui/01/05). PHA mitogen was used as a positive control. Cells (200,000 per well) were added to each well of the ELISPOT plates (Millipore, www.millipore.com), which had been coated with antiprimate IFN-γ antibody (Mabtech clone G2.4), and incubated at 4°C overnight. The wells were next washed six times with PBS to remove cells and were then treated with a biotinylated antiprimate IFN-γ monoclonal antibody (Mabtech clone 7-B6-1) in PBS containing 0.5% BSA for 2 h at room temperature. After another three washes with PBS, an avidin-alkaline phosphatase complex was added, followed by incubation for 1 h at room temperature. The wells were then washed, incubated with BCIP substrate (BioRad, www.bio-rad.com) for 15 min at room temperature, rinsed with distilled water to halt spot development, dried and read with an automated ELISPOT reader (AID, www.elispot.com). The number of spot-forming cells (SFCs) from each well was determined for each animal after subtraction of counts from cells cultured without peptide. A response was considered positive if SFCs exceeded 20 per 10^6^ PBMCs. The average response for negative peptides and mock controls was 3 SFC/million PBMC. The H5 HA response of each animal was taken the sum of the IFN-γ positive responses from all HA pools after subtraction of background counts.

To confirm the presence of peptide induced IFN- *γ* responses and their relationship to CD4+ or CD8+ T lymphocytes, we tested individual peptides from the first ELISPOT using a two-dimensional matrix system in a second ELISPOT assay, using thawed PBMC samples depleted of CD8+ T lymphocytes. Cell depletion was carried out with nonhuman primate CD8+-specific microbeads according to the manufacturer's instructions (Miltenyi Biotec, www.miltenyibiotec.com). CD8+ lymphocytes in the PBMC samples were removed by labeling the cells with specific microbeads in buffer containing PBS pH7.2, 0.5% BSA and 2 mM EDTA and then applying a magnetic field. Undepleted PBMCs were used as positive controls.

### Mouse study

Six-week-old female specific-pathogen-free BALB/c mice were used in this study. The wild-type and reassortant viruses were both tested for their replicative capacity, and the dose required to kill 50% of the mice (MLD_50_) was determined as described previously [1]. For the immunogenicity study, groups of 5 mice were anesthetized with CO_2_ before they were inoculated intranasally once or twice (4 weeks apart) with 10^6^ EID_50_ of the GSGD/AA*ca*, CKSX/AA*ca* or AH/AA*ca* virus. Sera were collected at 4 weeks after the first vaccination or second vaccination for the HI and NT antibody detection using the homologous wild-type H5N1 virus as antigen. For the vaccine study, 80 6-week-old female specific-pathogen-free BALB/c mice were anesthetized with CO_2_ before they were inoculated intranasally once or twice (4 weeks apart) with 10^6^ EID_50_ of the AH/AA*ca* in a 50 µl volume or with PBS as control. Serum samples were collected from six mice in each group at 4 weeks after the first and second immunizations and were examined for HI and NT antibody using the homologous AH/05 virus and heterologous BHG/05 virus as antigens. Four weeks after vaccination, the mice were challenged with 100 MLD_50_ of the AH/05 (10^3.5^EID_50_) or BHG/05 (10^3.6^EID_50_) virus intranasally; three mice from each group were killed on day 3 postchallenge, and their organs collected for virus titration. The remaining seven mice were observed for 15 days for body weight change and death.

### Macaque study

Sixteen female rhesus macaques (*Macaca mulatta*) 2 or 3 years of age were divided into two groups of eight animals each; one group (V1–V8) was inoculated intranasally twice (4 weeks apart) with 10^7^ EID_50_ of the AH/AA*ca* virus in a 1-ml volume, while the other (C1–C8) received the same volume of PBS as a control. Serum samples were collected from each animal at 2 and 4 weeks after the first immunization and 2 weeks after the second immunization. Three weeks after the second immunization, the animals in each group were challenged by intratracheal inoculation of 10^6^ EID_50_ of AH/05 virus (n = 4) or BHG/05 (n = 4) virus in a 3 ml volume. Three days later, two animals from each subgroup were euthanized, and different parts of their respiratory system were collected for virus titration and histologic and immunohistochemical examinations; the remaining animals were observed and euthanized on day 15 postchallenge. Nasal swabs were collected from all of the animals on days 2, 4 and 6 postchallenge for virus isolation in eggs.

### Pathologic examination

Tissues fixed in 10% phosphate-buffered formalin were dehydrated, embedded in paraffin, cut into 5-µm thick sections, and stained with standard hematoxylin-and-eosin. Immunohistochemistry was performed with antibodies to an H5 virus (A/Vietnam/1203/04) using the Dako Envision system (Dako, www.dako.com).

### Animal facility

Studies with highly pathogenic H5N1 avian influenza viruses inoculated into mice and macaques were conducted in a biosecurity level 3+ laboratory approved by the Chinese Ministry of Agriculture. All animal studies were approved by the Review Board of Harbin Veterinary Research Institute, Chinese Academy of Agricultural Sciences.

### Statistical analysis

Virus titers in mice, antibody titers of mice and monkeys were compared with a two-sided *t*-test.
